# Silent Witness: Dual-Species Transcriptomics Reveals Epithelial Immunological Quiescence to Helminth Larval Encounter and Fostered Larval Development

**DOI:** 10.3389/fimmu.2018.01868

**Published:** 2018-08-15

**Authors:** Friederike Ebner, Mathias Kuhring, Aleksandar Radonić, Ankur Midha, Bernhard Y. Renard, Susanne Hartmann

**Affiliations:** ^1^Department of Veterinary Medicine, Institute of Immunology, Freie Universität Berlin, Berlin, Germany; ^2^Bioinformatics Unit (MF1), Department for Methods Development and Research Infrastructure, Robert Koch Institute, Berlin, Germany; ^3^Core Unit Bioinformatics, Berlin Institute of Health (BIH), Berlin, Germany; ^4^Berlin Institute of Health Metabolomics Platform, Berlin Institute of Health (BIH), Berlin, Germany; ^5^Max Delbrück Center (MDC) for Molecular Medicine, Berlin, Germany; ^6^Center for Biological Threats and Special Pathogens: Highly Pathogenic Viruses (ZBS 1), Robert Koch Institute, Berlin, Germany

**Keywords:** host–pathogen, parasitic nematode, IPEC-J2, *Ascaris suum*, dual-species, RNA sequencing, transcriptomics, epithelial communication

## Abstract

Gastrointestinal nematodes are among the most prevalent parasites infecting humans and livestock worldwide. Infective larvae of the soil-transmitted nematode *Ascaris* spp. enter the host and start tissue migration by crossing the intestinal epithelial barrier. The initial interaction of the intestinal epithelium with the parasite, however, has received little attention. In a time-resolved interaction model of porcine intestinal epithelial cells (IPEC-J2) and infective *Ascaris suum* larvae, we addressed the early transcriptional changes occurring simultaneously in both organisms using dual-species RNA-Seq. Functional analysis of the host response revealed an overall induction of metabolic activity, without induction of immune responsive genes or immune signaling pathways and showing suppression of chemotactic genes like CXCL8/IL-8 or CHI3L1. *Ascaris* larvae, when getting in contact with the epithelium, showed induction of genes that orchestrate motor activity and larval development, such as myosin, troponin, myoglobin, and protein disulfide isomerase 2 (PDI-2). In addition, excretory-secretory products that likely facilitate parasite invasion were increased, among them, aspartic protease 6 or hyaluronidase. Integration of host and pathogen data in an interspecies gene co-expression network indicated links between nematode fatty acid biosynthesis and host ribosome assembly/protein synthesis. In summary, our study provides new molecular insights into the early factors of parasite invasion, while at the same time revealing host immunological unresponsiveness. Reproducible software for dual RNA-Seq analysis of non-model organisms is available at https://gitlab.com/mkuhring/project_asuum and can be applied to similar studies.

## Introduction

The large roundworms *Ascaris (A.) lumbricoides* and *Ascaris suum* are the most prevalent soil-transmitted helminths worldwide and parasitize the gastrointestinal tract of humans and pigs, respectively. With an estimated 800 million people currently infected, typically in the most impoverished populations, Ascariasis belongs to the group of neglected tropical diseases (1–3). Among pigs, the prevalence of *A. suum* in high intensity production farms remains high all over the world and *A. suum* infections are known to cause major economic losses in the pig industry due to reduced growth performance, liver condemnation, and reduced vaccination responses (4, 5). Due to the genetic closeness of *A. lumbricoides* and *A. suum* (6, 7), studying host–parasite interactions in pigs is not only of veterinary importance but also represents an ideal research model for the human condition (8).

Ascariasis is caused by ingesting infective eggs containing L3 larvae from contaminated food and water, or with regard to pigs, coprophagy (9). The eggs hatch in the intestine and parasites undergo larval migration through the body before developing into adult worms that inhabit the small intestine. The early, intestinal migratory path involves newly hatched L3 larvae penetrating the walls of the distal small intestine, cecum, and proximal colon (10, 11) and migrating toward the liver.

Given the initial invasion and chronic infestation at gastrointestinal barriers, the mucosal immune response is of fundamental importance for defending against the parasite. The first cells to encounter invading *Ascaris* larvae are epithelial cells. The intestinal epithelium represents a tight barrier that prevents pathogen invasion. However, rather than being just a physical barrier, epithelial cells are equipped with microbial-detection mechanisms, signaling circuits, and both homeostatic and inflammatory mediators (12, 13). At the interface between host and environment, the epithelium defends the host against infection to the one side while fostering innate immune recognition and transmission of danger signals to the other side (12). While many types of pattern recognition receptors (PRRs) facilitate sensing of microbes, it is less clear how enteric parasites like *Ascaris* spp., which do not express known PAMPs, are sensed and detected by the epithelium. Conversely, whether a targeted host–pathogen interaction at the epithelial barrier drives larval tissue migration is not yet known.

The site-specific mucosal penetration of invading *Ascaris* L3 occurs within 3–6 h after oral infection (11) and restricts the window for an early intervention. Infection and challenge studies have greatly contributed to our understanding of hepatic- and pulmonary immune responses against *A. suum* (11, 14, 15); however, not much is known concerning the initial steps of immune recognition and modulation during early larval migration. Understanding to what extend host and parasite might sense, interact, regulate, or harm each other at the epithelial interface might clarify how mucosal immunity is initiated and reveal possible intervention strategies.

Therefore, we performed a time-resolved transcriptional analysis of infective *A. suum* third-stage larvae (AscL3) co-incubated with porcine intestinal-epithelial cells (IPEC-J2), an *in vitro* model widely used to study microbial pathogen–host interaction (16, 17). In that context, RNA-Seq is extremely useful for addressing non-model organisms such as a parasitic nematode (*A. suum*) and the pig. Addressing the limitations of most current dual RNA-Seq pathogen–host studies, pointed out by Westerman and colleagues (18) in a recent review, we do not restrict our analysis to pairwise differential comparisons of sets of time points, but account for the complete temporal behavior. By contrasting splines fitted across the time lines to baseline models rather than individual time point comparisons, statistical power can be increased (19) and the statistical model can capture trends that otherwise may be missed due to the restricted temporal granularity of individual time points (18).

In contrast to intracellular microbial pathogens, the possibility of separating larvae and host tissue after incubation enables purer sequencing and thus transcriptome analysis is unaffected by asymmetric read coverage as well as cross read mappings. In addition, we complement annotation of the non-model *A. suum* transcriptome for functional analysis by implementing a unique iterative annotation strategy favoring transfer from closely related species. This workflow builds upon continuously increasing the search space of species, thereby favoring results from closely related species were available, but still annotating sequences were no hits on closely related species can be found.

While many software solutions for individual steps are available, we are not aware of any interconnected analysis pipeline for dual RNA-Seq studies of non-model organisms. Here, we rely on Snakemake (20) as a current bioinformatics community standard to provide workflows at https://gitlab.com/mkuhring/project_asuum/. These workflows not only allow full reproduction of our analyses but are provided for reusage in related experiments and capture all relevant steps. Particularly, we integrated and primarily automated the annotation steps for both non-model organisms with the differential expression and functional analysis.

Our time-resolved, dual-species whole-transcriptome approach provides molecular insights into host–parasite interactions during direct physical interaction and in the absence of surrounding microbes, their metabolites and second line immune cells, enabling us to focus exclusively on parasite-epithelial cross-talk. Our results demonstrate that *Ascaris* larvae invade the host soft-footed without initiating immune alarming responses while expressing genes ensuring invasion and their further development.

## Materials and Methods

### Generation of Infective *A. suum* L3 Stage Larvae and Hatching

Infective third-stage larvae of *A. suum* were generated as previously described (21). In brief, secreted *A. suum* eggs were collected from the culture fluid of female worms obtained from a local slaughter house. Eggs were decoated in 0.5 M sodium hydroxide, washed, and embryonated in 0.1 N H_2_SO_4_ for 6–8 weeks under weekly aeration. For hatching, embryonated eggs were treated with 5.25% sodium hypochlorite to remove the chitinous layer from the eggs. Hypochlorite-treated eggs were further layered below slowly moving sterile glass beads (4 mm) to facilitate hatching. Subsequently, intact and motile *A. suum* larvae were collected, washed, counted, and adapted to mammalian cell culture media (IMDM, 5% FCS, 1% P/S) over night.

### Parasite and Epithelial Cell Co-Incubation

Porcine intestinal epithelial cells (IPEC-J2 cell line) were cultured as monolayers for complete confluence in 35 mm Petri dishes [IMDM, 5% FCS, 1% P/S (all from PAN-Biotech, Aidenbach, Germany)]. L3 stage *A. suum* worms (50,000/dish, resulting in a larva to cell ratio of 1:10) were layered on top of epithelial cells and co-incubated for the indicated time points at 37°C, 5% CO_2_. Three biological replicates of a time-resolved co-incubation series of pathogen and epithelial cells were performed, with five time points (0, 1, 2, 3, and 9 h) being interrogated with regard to the early migratory pathway of *A. suum* (10, 11). Monocultures of either worms or epithelial cells served as controls (referred to as time point 0 h). Following co-incubation, *A. suum* larvae were removed from the epithelial layer and worms and epithelial cells were processed separately for RNA isolation. Viability of IPEC-J2 cells following larval coculture was verified in separate experiments by vital cell counting, measuring ATP as indicator for metabolically active cells and by documenting the monolayer appearance using Giemsa staining (Figures S6A–C in Supplementary Material). Asc L3 were visually inspected for larval motility throughout the entire coculture period and assayed for migration capacity and viability [excretory-secretory (ES) production] after 9 h of coculture (Figures S6E,F in Supplementary Material).

### RNA Isolation and Quality Check

Worm samples were homogenized using shredder columns filled with 200 mg sterile sea sand and the FastPrep^®^-24 instrument (MP Biomedicals) at 5 m/s for 35 s. Supernatants of homogenized worms and epithelial cell lysates were further processed for RNA isolation (InnuPREP RNA isolation, Analytik Jena AG, Germany), DNase treatment (Analytik Jena AG, Germany), and RNA quality control (Agilent 2100 Bioanalyzer, RNA 6000 Nano Kit, Agilent Technologies, Waldbronn, Germany). RNA sample RIN values ranged between 7.9 and 10 for porcine epithelial cells and 7.3 and 9.5 for AscL3, respectively.

### Sequencing and Mapping

For transcriptome sequencing on an Illumina platform a TruSeq RNA library generation was utilized. The library was generated by using the TruSeq RNA Sample Prep Kit v2 (Illumina, San Diego, CA, USA) following the manufacturer’s instructions. The library was quantified by using the KAPA Library Quantification Kit for Illumina (Kapa Biosystems, Wilmington, MA, USA). The library size was determined by using the High Sensitivity DNA Analysis Kit for the 2100 Bioanalyzer Instrument (Agilent Technologies, Waldbronn, Germany). Libraries were adjusted to a concentration of 12 pM and sequenced on a HiSeq 1500 instrument (Illumina, San Diego, CA, USA) in high-output mode. For cluster generation, the TruSeq PE Cluster Kit v3 was used. Cluster generation was performed on a cBot instrument. For sequencing, the TruSeq SBS Kit v3 was used to sequence 100 + 100 bases.

**Figure 1:**
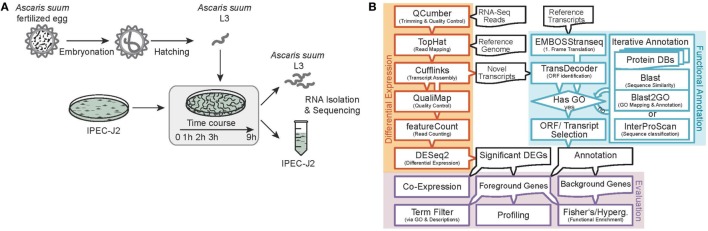
Experimental design and RNA-Seq data processing. **(A)** Analysis of the early host–parasite interaction of *Ascaris suum* L3 and porcine epithelial cells (IPEC-J2) by studying dual-species gene expression dynamics over a time course (0–9 h). **(B)** Paired RNA samples were Illumina sequenced, preprocessed, mapped to reference genomes, annotated, and differential gene expression and functional analysis was performed using the illustrated bioinformatics pipeline.

Sequence data processing and analysis were conducted as illustrated in Figure 1B. We sequenced 15 (resulting in three replicates for each of the five time points) AscL3 and IPEC-J2 samples each, with a mean library size of 45.5 million paired-end reads and a SD of 25.4. Raw reads were subjected to quality control and trimming *via* the QCumber pipeline (version 1.0.0)^1^ utilizing FastQC (v0.11.5)^2^, Trimmomatic (0.33) (22), and Kraken (0.10.5-beta) (23). On average, 88.21% of reads remained after trimming.

Preprocessed reads were mapped to reference genomes as specified below and corresponding sequence features using the TopHat split-read mapper (v2.1.1) (24) and reference as well as novel features were extracted and merged with the aid of Cufflinks (25) and Cuffmerge (25) (v2.2.1) to obtain one integrated and unified transcriptome for AscL3 and IPEC-J2 samples, respectively. Reference genomes used included the original *A. suum* draft genome from Jex et al. (26) as available from the WormBase ParaSite (27) FTP server (ftp.wormbase.org/pub/wormbase/species/a_suum/assemblies/v1/) and the *Sus scrofa* genome assembly from Ensembl (28) (Sscrofa10.2, release 85).

### Differential Expression

For each sample, raw expression values were created by counting uniquely mapped reads on gene level using featureCounts (v1.5.0-p3) (29). To identify differentially expressed genes (DEGs) within AscL3 and IPEC-J2 samples, respectively, DESeq2 (1.12.4) (19) was adjusted for a time series design model based on natural splines to account for the time points in the experiment. A likelihood-ratio test against fits of a reduced null model including only intercept and the batch variable was applied to infer significant DEGs. However, pairwise fold-changes for each time point greater than 0 h were extracted *via* a classic DESeq2 model and pairwise contrasts with the base time point 0 h (indicating expression changes in comparison to 0 h). In addition, normalized and transformed expression values were extracted from DESeq2 (regularized log transformation) and corrected for batch effects *via* Limma (3.28.21, removeBatchEffect) (30) to allow for sample quality control with clustered heatmaps and principal component analysis (PCA).

### Functional Annotation

Reference as well as novel transcripts were functionally annotated with focus on Gene Ontology (GO) terms (31, 32) using a novel iterative annotation strategy. First, transcripts were either first-frame translated (reference) or examined for ORFs (novels, Cuffcompare class code “u”) using EMBOSS transeq (6.6.0.0) (33) and TransDecoder (v2.1)^3^, respectively. Next, resulting protein sequences were passed through a series of database searches until successfully annotated with GO, either *via* blastp (2.6.0+) (34) and Blast2GO (4.0.7) (35) or by a final InterProScan classification (5.23–62.0 for *A. suum*, 5.22–61.0 for *S. scrofa*) (36). Databases used for *A. suum* sequences annotation included (in this order) the UniProt (37) *A. suum* proteome (UP000017900), UniProt Swiss-Prot Nematoda proteins, UniProt TrEMBL Nematoda proteins as well as the complete Swiss-Prot database and the complete TrEMBL database (all downloaded at 16.02.2017). For *S. scrofa* sequences, Ensembl reference annotations were obtained *via* the Ensembl BioMart server (38, 39) (with date of 30.10.2016). In addition, remaining non-annotated transcripts were searched against the UniProt *S. scrofa* proteome (UP000008227), Swiss-Prot Mammalia proteins, TrEMBL Mammalia proteins (all downloaded at 27.02.2017) as well as the complete Swiss-Prot database and the complete TrEMBL database (both downloaded at 16.02.2017).

In addition to GO terms, protein sequences were annotated with KEGG Orthology terms (KO) (40) *via* the BlastKOALA web interface (2.1) (41) using the “family_eukaryotes” and “genus_prokaryotes” databases for *A. suum* sequences and the “genus_eukaryotes” database for *S. scrofa* sequences, respectively (database builds 02.05.2017). However, for *S. scrofa*, reference annotations obtained *via* biomaRt (2.28.0) (42) and KEGGREST (1.12.3)^4^ were preferred if available. KO terms were then mapped to KEGG pathways *via* KEGGREST. Furthermore, *A. suum* sequences were examined for potential excretory and secretory proteins as previously described by others (43). In brief, ES proteins were annotated as classical or non-classical secretory proteins by combining SignalP (4.1 Server, organism group “Eukaryotes”) (44) and SecretomeP (2.0 Server, organism groups “Gram-negative bacteria,” “Gram-positive bacteria,” and “Mammalian”) (45, 46). Mitochondrial proteins were excluded from that list using TargetP (1.1 Server, organism group “Non-plant”) (47) as well as proteins predicted for transmembrane regions by TMHMM (Server v. 2.0) (48). The remaining predicted ES proteins were further categorized by KEGG ENZYME enzyme codes derived from KO-terms with the use of KEGGREST.

### Functional Analysis

Functional profiles and enrichment analysis of significant DEGs were calculated based on GO term annotations (projected onto level 2) of the three classes of molecular function (MF), cellular component (CC), and biological process (BP) as well as on KEGG Pathways. For enrichment analysis, significant DEGs were tested against background gene sets representing the expression potential in the experiment, i.e., all expressed and sufficiently annotated genes in the AscL3 and IPEC-J2 samples, respectively. Significantly over- and underrepresented GO terms were determined by the two-sided Fisher’s Exact test in Blast2GO (adjusted *p* < 0.05) while enriched KEGG pathways were determined with a hypergeometric test in clusterProfiler (3.0.5) (49) (adjusted *p* < 0.05). In addition to global analysis, significant DEGs were mined for specific functions of particular interest *via* filters based on specifically selected GO terms, free-text searched GO term collections, as well as gene description analysis. Thereby, GO term filters were recursively extended with corresponding child terms to account for descending functionality using GO.db (3.3.0)^5^. Target functions as well as corresponding GO-terms and description filter are found in Table S3 in Supplementary Material.

### Co-Expression Analysis

The normalized, transformed, and batch-corrected expression values were used to infer an interspecies gene co-expression network (GEN) between *A. suum* and *S. scrofa*. The AscL3 and IPEC-J2 expression matrices were combined (excluding replicate C of time point 2 h due to quality issues) and subjected to pairwise Spearman rank correlation tests using the psych R package (1.7.5) (50) with FDR *p*-value adjustment for multiple testing. Resulting gene pairs (edges in the GEN) were filtered by significance (adjusted *p* < 0.05) and correlation strength (rho > 0.95) before further analysis and visualization within Cytoscape (3.2.1) (51). In Cytoscape, duplicated edges and self-loops were removed and the network was reduced to nodes participating in interspecies interactions (GEN1). Node degrees were calculated based once on all edges and once on interspecies edges alone (GEN2) to identify highly interacting genes between species. In addition, node (resp. gene) clusters were calculated on GEN1 with the MCODE plugin (v1.4.1) (52).

## Results and Discussion

### Transcriptional Response Reveals Temporal Dynamics of Early Host–Pathogen Interaction

A critical event during initial invasion of *Ascaris* parasites is crossing the intestinal epithelial barrier of their mammalian hosts. The initial dialog of host epithelium and parasite before the parasite starts tissue migration has, however, received little attention. We thus performed a time-resolved RNA-Seq transcriptional profiling of both, *A. suum* L3 larvae and porcine epithelial cells (IPEC-J2) to examine the early and comprehensive transcription of genes potentially involved in sensing, attachment, barrier disruption, and immune response early after initial parasite–host contact.

We sequenced *A. suum* larvae (AscL3) and IPEC-J2 at five different time points during co-incubation (0, 1, 2, 3, and 9 h) with *n* = 3 biological replicates each (Figure 1A). Mapping quality control of raw reads using QualiMap (v.2.2.1, Figure 1B) (53) indicated one sample (IPEC-J2, 2 h, replicate C) being inconsistent in comparison to all the other samples in the experiment, which consequently was excluded from further analysis.

**Figure 2:**
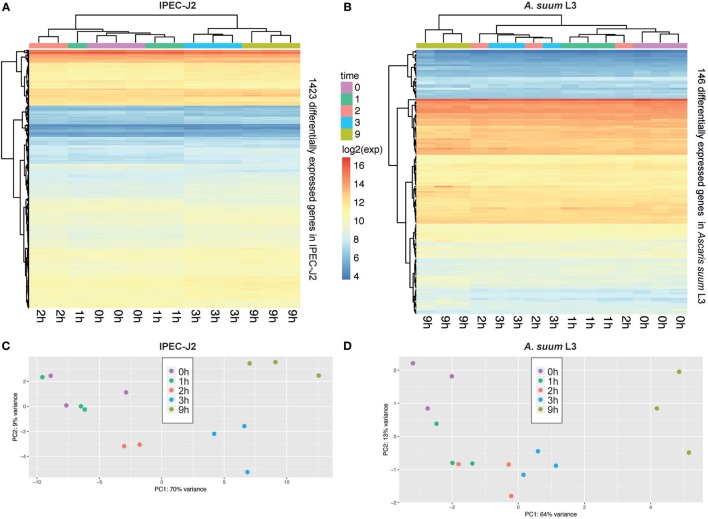
Principal component analysis (PCA) of porcine epithelial cells (IPEC-J2) and *Ascaris suum* L3 expression data and clustering of differentially expressed genes (DEGs). **(A)** Unsupervised clustering heatmap of DEG in porcine epithelial cells (*n* = 1,423, IPEC-J2) and **(B)** *A. suum* L3 (*n* = 146). Red intensity indicates high gene expression during the time-course of host–parasite co-incubation, whereas blue intensity indicates low gene expression. PCA of RNA-Seq samples of **(C)** porcine epithelial cells and **(D)** *A. suum* L3 reveals that 79% (IPEC-J2) and 77% (*A. suum*) of the data variation is explained by the first two PCs, respectively. The five time points are color-coded (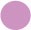 0, 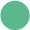 1, 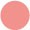 2, 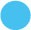 3, and 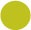 9 h) and comprise *n* = 3 biological replicates (exception: *n* = 2 for IPEC-J2; 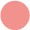 2 h, due to RNA-Seq quality control exclusion).

In total, our exploratory study identified 146 *A. suum* genes and 1,423 *S. scrofa* genes with significant differential expression over time (Table 1, with adjusted *p*-value < 0.05 for *A. suum* and 0.01 for *S. scrofa*, respectively). Normalized expression values of both IPEC-J2 and *A. suum* L3 genes demonstrated successful clustering of replicates by time with minor exceptions of earlier time points for IPEC-J2 (Figure 2A) and median time points for AscL3 (Figure 2B). Clustering analysis indicates that most distinct changes in expression occur in later time points, additionally confirmed by the pairwise fold-changes (Table S1 in Supplementary Material). PCA of IPEC-J2 (Figure 2C) and *A. suum* L3 (Figure 2D) expression data indicate a relation of the first principal component with time direction and thus confirms that most of the variance and, therefore, change in expression is occurring over time.

**Table 1:** Numbers of differentially expressed and annotated genes.

	Total DEG	FC ≥ 1.5^a^	FC ≥ 1.8^a^	FC ≥ 2^a^
**Complete**				
*Ascaris suum* (α = 0.05)	146	74	17	9
*Sus scrofa* (α = 0.01)	1,423	763	181	56
**GO-annotated**				
*A. suum*	87	34	11	5
*S. scrofa*	1,214	621	121	33

*^a^abs(log(fold-change)) ≥ log(*n*)*.

*Total and gene ontology (GO) annotated numbers of differentially expressed genes (DEG) and DEG numbers for distinct fold-change cut-offs (with respect to the maximal pairwise fold-change) are depicted for *A. suum* L3 and *S. scrofa* epithelial cell (IPEC-J2) expression data*.

Particularly for genes of the non-model nematode *A. suum*, available GO annotation was limited. We, therefore, implemented an iterative annotation strategy, depicted in Figure 1B and Figure S1 in Supplementary Material, which enabled us to assign GO terms to 13,683 reference and 410 novel *A. suum* genes as well as to 21,265 reference and 890 novel *S. scrofa* genes (including Ensembl reference GO-annotations). Regarding our co-incubation experiment, 59.6% of the *A. suum* DEGs (87 of the 146) and 85.3% of the *S. scrofa* DEGs (1,214 of the 1,423) were adequately annotated and thereby eligible for further functional analysis (Table 1). In parallel, a total of 8,034 *A. suum* genes could be annotated with KEGG Orthology (KO) identifiers including 7,306 reference and 728 novel genes. Furthermore, 9,711 *S. scrofa* genes could be annotated with KOs comprising 8,954 reference and 757 novel genes. KEGG pathways were assigned to 59 *A. suum* and 471 *S. scrofa* DEGs, respectively. These data highlight the GO annotation gap for *A. suum* genes and point out a need for improving methods to functional annotate nematode genes in general. A complete list of all *A. suum* L3 and IPEC-J2 genes (annotated and not annotated) is provided as Table S4 in Supplementary Material.

### The Epithelial Cell Response to Worm Invasion

#### Epithelial Cells Are Not Intensely “Alarmed”

**Figure 3:**
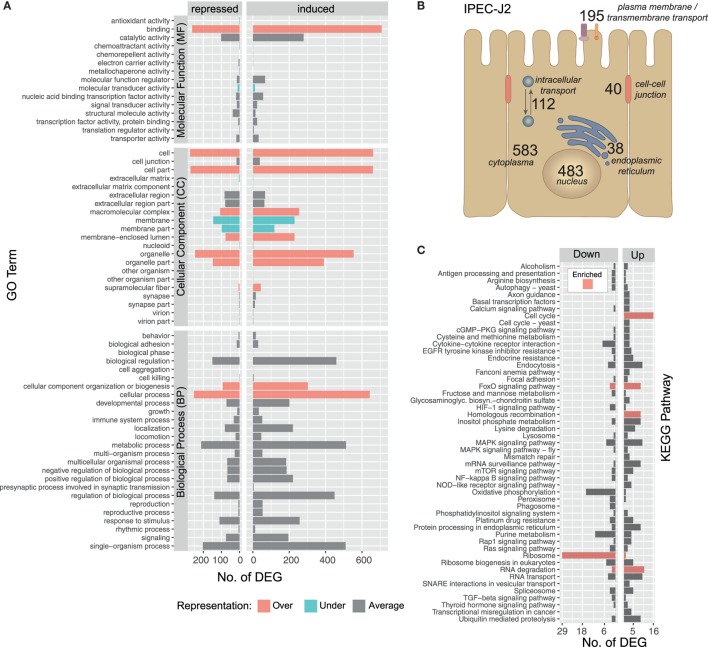
Gene ontology (GO) and pathway profiling of porcine intestinal epithelial cell differentially expressed genes (DEGs). An iterative annotation strategy was used to assign GO terms to reference and novel porcine transcripts. **(A)** For 1,214 GO-annotated porcine DEGs, the level 2 of GO hierarchy is illustrated and classified into Molecular Function (MF), Cellular Component (CC) and Biological Process (BP). GO terms significantly enriched among differentially expressed *S. scrofa* genes (Fischer’s Exact Test, two-sided, *p* < 0.05) are highlighted as over-(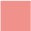) and under-(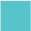) represented terms alongside with averagely represented terms (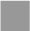) with regard to the reference genome. **(B)** Subcellular localization of DEG encoding proteins deduced from binning relevant GO terms (Table S3 in Supplementary Material) and offsprings [*nucleus* (CC, 483), *endoplasmic reticulum* (CC, 38), *cytoplasm* (CC, 583*), intracellular transport* (BP, 112), *plasma membrane* (CC, 128), *transmembrane transport* (BP, 34), *transmembrane transporter activity* (MF, 33), *cell–cell-junction* (CC, 31), and *cell–cell junction organization* (BP, 9)]. **(C)** Sequences were annotated with KEGG pathways and pathways with three or more assigned DEGs are visualized including color-coded (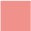) enrichment analysis (hypergeometric test, *p* < 0.05).

The IPEC-J2 cell line is a non-transformed, porcine epithelial cell line derived from the small intestine and currently the most convincing model for porcine infection studies (16, 54). The initial contact of *A. suum* larvae with the intestinal epithelial cells over 9 h results in a transcriptomic response of 1,423 *S. scrofa* genes (982 upregulated/441 downregulated, with respect to the maximal pairwise fold-change). To functionally describe the regulated *S. scrofa* genes, level 2 GO term profiles are illustrated (Figure 3A), categorizing MF, CC, and BP. To gain insight into the biological significance of alterations in gene expression levels, GO enrichment analysis was used to determine whether certain GO terms are over- or underrepresented within the gene set of interest. GO terms significantly overrepresented (adjusted *p* < 0.05) among *S. scrofa* DEGs were *binding* (as part of MF), *CC organization* or *biogenesis and cellular process* (BP) as well as several CC including *cell, cell part, macromolecular complex, membrane-enclosed lumen, organelle, organelle part*, and *supramolecular fiber* (Figure 3A). Surprisingly, the few underrepresented terms are almost exclusively attributed to extracellular matrix (ECM), membrane, and membrane part (CC), a group of terms that we expected to be specifically addressed while being co-incubated with a comparably large and motile extracellular parasite. Further global analysis as subcellular location of *S. scrofa* DEGs and KEGG pathway annotation are depicted in Figures 3B,C. *S. scrofa* KEGG profiling features several significantly enriched pathways (hypergeometric test, adjusted *p* < 0.05) including induced cell cycle activity, FoxO signaling, homologous recombination and RNA degradation as well as a suppressed ribosome pathway. In addition, though not significant, oxidative phosphorylation and purine metabolism show distinct repression (Figure 3C). Genes of the FoxO family that are central for the here enriched *FoxO signaling* pathway regulate transcriptional responses including apoptosis, cell-cycle control, glucose metabolism, oxidative stress resistance, and longevity. A recent study revealed that intestinal FoxO-mediated signaling is required for epithelial antimicrobial response and AMP synthesis by enterocytes (55). Factors inducing FoxO signaling include oxidative and nutrient stress stimuli, such as insulin or several growth factors. The induced oxidative stress response is in line with the repression of the oxidative phosphorylation pathway, and together imply increased nutrient consumption or even nutritional competition between IPEC-J2 and *A. suum* larvae.

KEGG pathway analysis further highlights that pathways linked to extracellular recognition such as TLR signaling are not regulated (Figure 3C). This is supported by several other studies showing that host PRRs such as TLR are modulated by nematode species to limit inflammation (56–58) but TLR signaling in generating an anti-helminth immune response remains controversial (59). Since epithelial cells are known to express all kinds of PRRs, we more specifically considered the role of C-type lectin receptors such as mannose receptor, DC-sign, ICAM-3, collectins, or selectins (59, 60) to be addressed, but found no transcriptional evidence that these receptors or corresponding receptor signaling pathways are affected by the presence of *A. suum* larvae. Interestingly, we observe a generally low presence of *S. scrofa* DEGs in several other signal transduction pathways, such as HIF-1, MAPK, mTOR, NF-kappa B, Rap1, TGF-ß, or RAS indicating that IPEC-J2 cells overall were not in an “alarmed state” as expected.

**Figure 4:**
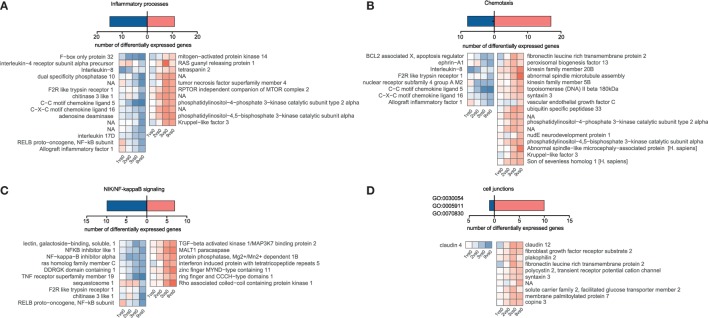
Temporal gene expression changes in porcine intestinal epithelial cells in response to *A. suum* larvae. Blastp, Blast2GO, and InterProScan were used to identify *S. scrofa* differentially expressed genes associated with distinct immune responses or cell junction for **(A)** “Inflammatory processes,” **(B)** “Chemotaxis,” and **(C)** “NIK/NF-kappaB signaling.” For **(D)** “Cell junctions,” the cell–cell junction localization filter was manually selected for GO:0030054, GO:0005911, and GO:0070830.

Although we hypothesized that epithelial cells somehow sense incoming *Ascaris* larvae and might orchestrate an appropriate response, we were surprised to find such a low magnitude of response (indicated by fold-change values) and the lack of conclusive activation signatures. We then specifically looked for genes involved in inflammatory responses (Figure 4A, GO declination in Table S3 in Supplementary Material) and chemotactic responses (Figure 4B, GO declination in Table S3 in Supplementary Material) and found chemokine transcripts (CCL5, IL8, and CXCL18), the chitinase 3 like 1 protein, and the cytokines genes for IL17D to be suppressed. Interleukin-8, one of the repressed immunity genes, is a potent chemoattractant for primary neutrophils but also other granulocytes, and is secreted by epithelial cells in response to bacterial stimuli (61). Upon LPS stimulation, also porcine IPEC-J2 cells rapidly upregulate IL-8 transcripts (Figure S6D in Supplementary Material). Intriguingly, Aprianto and colleagues (62) showed that a highly adherent, unencapsulated form of *S. pneumoniae* suppressed IL-8 production from epithelial cells compared to a free-floating, non-adherent form of the same strain early during infection. Their RNA-Seq study thereby links repression of epithelial innate immune response with the adherence mechanisms of the invading pathogen and provides evidence for targeted immune evasion. Hence, active suppression of the innate chemokine response combined with restricted epithelial recognition of AscL3 larvae, weak induction and even suppression of NIK/NF kappaB signaling (Figure 4C), could explain the poor response of immune-related genes that we observed. Following this line, mice lacking NF kappaB signaling specifically in epithelial cells are incapable of mounting protective type 2 cell-mediated immunity to the nematode *Trichuris muris* in its rodent host (63). The initiation of protective type 2 responses against helminth typically begins with production of epithelial cell-derived cytokines (IL-25, IL-33, and thymic stromal lymphopoietin), but none of them was found to be regulated in porcine IPEC-J2 cells early after *A. suum* co-incubation. However, it has to be taken into account that it was recently found that rather than every epithelial cell, a very rare epithelial cell type, intestinal tuft cells, initiated mucosal type 2 responses to helminth parasites through IL-25 secretion after TRMP5 taste chemoreception (64, 65).

Studies in the mouse model parasitic nematode *Heligmosomoides polygyrus* showed that factors secreted by the nematodes directly suppressed IL-33 release (66), but the upstream mechanisms of IL-33 release are somewhat controversial and suggest that IL-33 might also be released from intracellular stores when cells undergo necrosis or even after mechanical stress (67). However, from our data, we cannot conclude that IL-33 is not released. We, therefore, speculated that during co-incubation, worms cause epithelial barrier damage and induce mechanical stress responses or cell death, but the latter was only poorly reflected in the epithelial transcriptome response [no wound healing, apoptosis, or necroptosis pathways (Figure 3C)]. From 247 DEGs associated with stress response in general (GO declination Table S3 and Figure S4 in Supplementary Material), only 10 are connected to mechanically induced stress responses/mechanical stimulus (Figure S5 in Supplementary Material). However, when genes associated to cell junction or bicellular junction formation (GO:0030054, GO:0070830, and GO:0005911) were considered, we noticed a clear trend toward an induced gene response, including claudin-12, plakophilin 2, and FLRT3 (Figure 4D). This highlights an active response to promote barrier integrity of IPEC-J2 and raises the question whether *A. suum* larvae and/or their secreted products specifically target tight junction formation. Moreover, the only downregulated gene in this functional group was claudin-4 (Figure 4D). This is particularly interesting, because researchers suggest that claudin-4 acts to tighten the paracellular pathway (68) and downregulation of claudin-4 is observed under conditions leading to increased permeability (69). The differential regulation of claudin-12 and claudin-4 and the downregulation of IL-8 was additionally validated by quantitative PCR (Figure S7A in Supplementary Material).

Together, our results indicate that small intestinal epithelial cells show poor innate immune responses to co-incubated *A. suum* L3, reduced ribosome function and oxidative phosphorylation pathways, but specifically induce tight junction formation. Whether IPEC-J2 cells do not possess an adequate detection system or whether the parasite is inactivating the host detection systems that would otherwise raise the alarm remains unknown.

### The Worm Response to the Epithelial Barrier

#### *Ascaris suum* Larvae Drive Invasion and Migration

**Figure 5:**
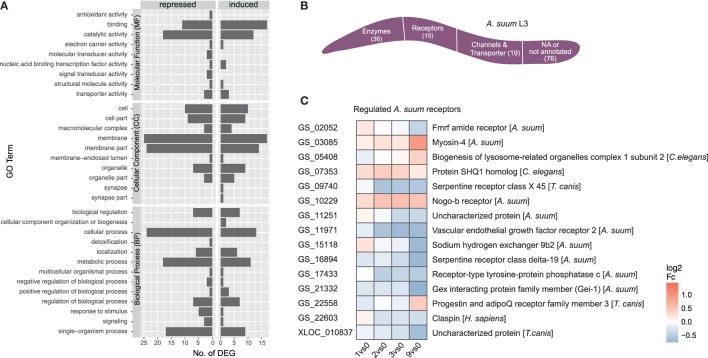
Gene ontology (GO) profiling of *Ascaris suum* differentially expressed genes (DEGs). **(A)** Distribution of level 2 GO terms for *A. suum* L3 DEGs, categorized into molecular function, cellular component (CC), and biological process (BP). **(B)** Global classification of *A. suum* L3 DEGs into enzymes, receptors, and channels/transporter based on GO-term filter and gene description analysis (Table S3 in Supplementary Material). **(C)** Time-resolved expression of *A. suum* DEGs with GO assigned receptor function.

The initial contact of *Ascaris suum* L3 with intestinal epithelial cells resulted in a transcriptomic response of 146 *A. suum* genes (68 upregulated/78 downregulated, with respect to the maximal pairwise fold-change). A total of 87 genes mapped to GO terms showed regulated functions such as *binding* and *catalytic activity* (MF), *membrane and membrane part* (CC) as well as *cellular, metabolic*, and *single-organism processes* (BP) (Figure 5A GO level 2). However, no significant enrichments (two-sided Fisher’s Exact test in Blast2GO; adjusted *p* < 0.05) could be observed with respect to the background genome. Due to the small number of *A. suum* DEGs in general, only a few KEGG pathways could be assigned more than once, namely ABC transporters (2), cAMP signaling pathway (2, repressed), cysteine and methionine metabolism (2, repressed), glycosaminoglycan degradation (2, activated), protein digestion and absorption (3), pyrimidine metabolism (2), RNA degradation (2), and spliceosome (2) (Figure S2 in Supplementary Material). GO term filtering and gene description analysis were used to classify *A. suum* L3 DEGs into enzymes (36), receptors (15), and channels/transporters (19) (Figure 5B). Surprisingly, most receptor associated genes were indeed downregulated (10 out of 15, Figure 5C). We specifically looked for *Ascaris* C-type lectins (C-TL), a superfamily potentially involved in either site-specific tissue recognition or interference with mammalian C-TL-mediated inflammation (70), but found no C-TL within the *A. suum* DEGs of our early response experiment.

In contrast, the 20 most upregulated *A suum* L3 genes (Table S1 in Supplementary Material) included genes associated with motor activity (GS_03085 Myosin-4, GS_04138 Troponin T, GS_00138 Myoglobin) and structure (GS_04352 Cuticle collagen domain-containing protein, GS_11610 Cuticle collagen 6, GS_09547 Cuticle collagen 34, GS_00653 Protein disulfide-isomerase 2). The upregulation of genes associated with motor activity/motility driven by epithelial-contact is particularly interesting in the absence of host peristalsis, the luminal content/microbes, or mucus production and could be indicative of increased locomotion either in order to penetrate host tissue or to counteract passive movement after site-specific recognition. Increased muscular locomotor activity together with proteolytic enzymes secreted by glandular structures are thought to be essential for host tissue penetration.

The subset of upregulated genes involved in cuticle formation such as protein disulfide-isomerase 2 (PDI-2) (Table S1 in Supplementary Material and qPCR-validated in Figure S7B in Supplementary Material) suggests an epithelial trigger for driving further larval development. Nematode cuticles are formed by multiple collagenous layers that contain extensive disulfide linkages (71) and new cuticles are synthesized sequentially for each developmental stage. Cuticle biogenesis in the endoplasmic reticulum involves proline hydoxylation and disulfide bond formation by PDI (72). Mutations in the Protein disulfide-isomerase 2 (PDI-2) gene of *Caenorhabditis elegans* resulted in severe body morphology defects, uncoordinated movement, adult sterility, abnormal molting, and aberrant collagen deposition (73). The importance of PDI-2 activity for ECM formation demonstrated for *C. elegans* and more recently also for *Brugia malayi* (74) highlights its relevance as a potential anthelmintic drug target (72), including for *Ascaris* species.

**Figure 6:**
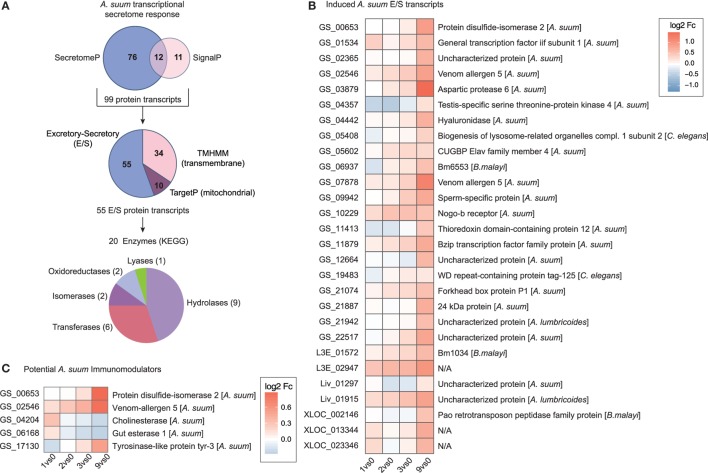
The secretome response of *Ascaris suum*. **(A)** The pipeline to identify *A. suum* excretory-secretory (ES) transcripts used SignalP and SecretomeP to annotate classical (*n* = 23) and non-classical (*n* = 88) ES genes and excluded mitochondrial (*n* = 10; TargetP) and transmembrane (*n* = 34; TMHMM) protein transcripts. KEGG ENZYME identified enzyme classes of ES transcripts are depicted in the bottom pie chart. **(B)** Time-resolved expression of *n* = 28 induced *A. suum* ES transcripts. **(C)** Differentially expressed *A. suum* transcripts that matched to genome prediction of *A. suum* immunomodulatory ES products based on homology to other nematodes (26).

At the host–parasite interface, ES proteins released by helminths play an important role in mediating host–parasite interaction, regulation, and control (75–78). To assess the transcriptional regulation of proteins potentially secreted by larval *Ascaris* parasites, we merged predicted DEGs for classical (SignalP) and non-classical (SecretomeP) secretory proteins and removed mitochondrial (TargetP) and transmembrane (TMHMM) proteins (Figure 6A). From the 55 ES proteins, 20 can be assigned to enzymatic activity with diverse functions including hydrolases (*n* = 9), transferases (*n* = 6), isomerases (*n* = 2), oxidoreductases (*n* = 2), and lyases (*n* = 1) as depicted in Figure 6A.

We hypothesized that proteolytic enzymes would be essential for parasite invasion and, therefore, upregulated by *A. suum* larvae once the epithelial barrier was detected. Within the class of proteases, nematode serine proteases have received considerable interest as they are widely distributed in parasitic nematodes with a wide variety of functions (79). Indeed, the parasite transcript with the highest maximal fold-change (comparing 0 versus 9 h time point, Table S1 in Supplementary Material) was identified as being *A. suum* aspartic protease 6 (Figure 6B). Aspartic proteases are a group of endopeptidases characterized by their catalytic aspartic residues that are known to drive host hemoglobin digestion by the blood-feeding nematodes *Haemonchus contortus, Ancylostoma caninum*, and *Necator americanus* (80, 81). Moreover, aspartic protease 1 from *N. americanus* is targeted by the bivalent human hookworm vaccine [Na-APR-1(M74), carrying a site-directed mutation abolishing its catalytic activity, and glutathione *S*-transferase (Na-GST-1)] currently being tested in clinical phase 1 studies in the US and Brazil (82–84). The Na-APR-1(M74) vaccine strategy is based on the induction of neutralizing antibodies against aspartic protease 1, thereby preventing hemoglobin degradation in the brush border membrane of the parasite’s digestive tract and finally leading to diminished parasite-related blood loss and reduced numbers of hookworms [reviewed in Ref. (83, 85)]. For nematode parasites that do not feed on blood but express aspartic proteases, different functions such as skin macromolecule and epithelial degradation, aiding in tissue penetration, or host-derived nutrient digestion are suggested (86–88). Interestingly, a proteomic study on ES products of different larval stages of *A. suum* shows the presence of aspartic protease 6 protein in ES collected from L3-lung stage worms, but its absence in the ES of hatched L3 larvae (89), thereby directly supporting our findings on the protein level and highlighting an epithelial trigger and early role for this specific enzyme.

The same study revealed the high abundance of glycosyl hydrolases (family 31, GH31) in *A. suum* L4 ES (89) suggesting degradation of complex carbohydrates to be essential for its energy metabolism. Among the ES enzymes upregulated directly in response to epithelial cell contact, we indeed identified a hyaluronidase (Figure 6, GS_04442). There is evidence that some bacterial species use hyaluronidases to utilize host hyaluronic acid (HA), an essential part of the ECM of epithelial tissue, as a carbon source for their energy metabolism (90). This might indicate that *A. suum* L3 not only produce proteolytic enzymes that help in epithelial tissue invasion but that epithelial ECM components can be directly hydrolyzed to feed on. Moreover, the release of hyaluronidase by *A. suum* has been described earlier for larvae isolated from the lungs of infected pigs (91). Besides its role in facilitating larval migration, the authors added another functional point: the modulation of developmental processes by hydrolyzing HA present in extracellular cuticle during molting. The role of *A. suum* hyaluronidase in either breaking up the internal cuticle-epidermal connection critical for molting (91), larval migration (92), or larval energy metabolism, therefore, remains speculative.

A comparison of the herein identified *A. suum* DEGs with a draft genome-based list of potential *A. suum* immunomodulators (26) revealed three upregulated transcripts (Figure 6C, GS_00653 PDI-2, GS_02546 venom allergen-5 and GS_17130 tyrosinase like protein tyr-3). Genomic and proteomic evidence suggest the abundance of venom allergen-like (VAL) proteins (SCP/TAPs superfamily) also in the ES compartment of *A. suum*. The early upregulation of VAL genes (venom allergen-5: qPCR validated in Figure S7B in Supplementary Material) is perhaps not surprising and has been associated in previous studies with larval invasion (78, 93, 94), although there is only limited understanding on the detailed function of those proteins in the host–parasite relationship.

In summary, we provide transcriptomic evidence for an early response pattern of genes that *A. suum* L3 specifically regulate during contact with host epithelial cells. Among those genes are factors that might facilitate parasite invasion through the epithelial barrier (aspartic protease 6), migration (myosin, troponin, and myoglobin), feeding (hyaluronidase), and development (PDI2) of the parasite. However, the parasite–host interaction not only depends on proteins but might also include glycans, lipids, miRNAs, or other small molecules and metabolites that have not been studied here, but can also contribute to parasite-epithelial communication (75). Furthermore, intestinal parasites and microbes have co-evolved together in their respective hosts. For that reason, the complex interaction of parasites with the bacterial community and the microbiota–host interaction likely also contributes to the parasite–host communication.

### Interspecies Interaction Is Dominated by Four *A. suum* Genes

Inference of interspecies gene co-expression networks (GENs) is an important systems biology approach to predict pathogen–host interactions. We applied established and efficient procedures to infer and analyze GENs including pairwise correlation, cluster (module) analysis, and functional enrichment (95, 96). Thereby, genes featuring a similar expression profile over time are associated by direct network links as well as gene clusters and are considered to participate in common functionality or in concurrent and thus interlinked processes. It must be noted that the recommended sample number of 20 is not met; however, our analysis compensates with distinctly higher read coverage than recommended (>10 million), an encompassing context of all samples due to the time series as well as strict GEN creation parameters (*p* < 0.05, rho > 0.95), resulting in a few but prominent and explicit signals.

**Figure 7:**
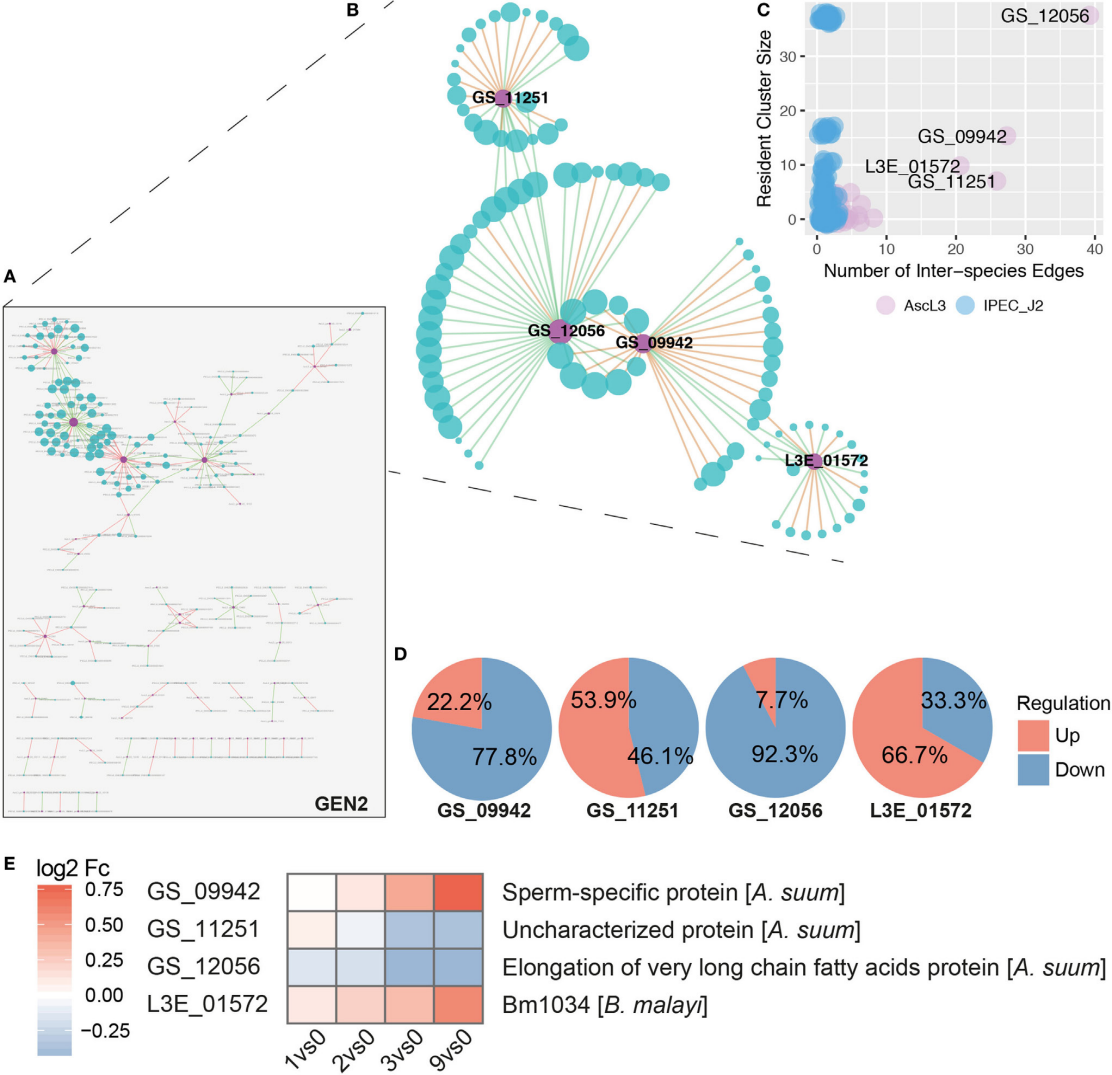
Inferred interspecies gene co-expression network. **(A)** Gene co-expression network reduced to interspecies nodes and interspecies edges (GEN2). Nodes represent co-expressed genes of *S. scrofa* (blue) and *A. suum* (violet) with node sizes corresponding to node degree (number of node interactions in GEN1). Edges between two nodes represent correlations that are either positive (green) or negative (red) with a Spearman correlation > 0.95. **(B)** The four most prominent interspecies clusters (Cluster 1, 2, 4, and 5, Table S2 in Supplementary Material) of the interspecies network GEN1 projected to GEN2 (i.e., without intra-species edges). **(C)** Number of interspecies edges versus cluster size for *A. suum* (AscL3, violet) and *S. scrofa* (IPEC-J2, blue) nodes [differentially expressed genes (DEGs)]. **(D)** Averaged gene regulation direction (based on max Fc) for all *S. scrofa* (IPEC-J2) DEGs connected to the four indicated *A. suum* (AscL3) genes. **(E)** Annotation and gene expression of the four interspecies network dominating *A. suum* genes over time by heatmapping the log2 Fc.

Based on node degrees of the co-expression network that was reduced to interspecies nodes and interspecies edges (Figure 7A, GEN2), we identified four highly interacting *A. suum* genes (Figures 7B,C). Three of the four highly interacting *A. suum* genes could be annotated by the iterative strategy (Figure 1B); GS_12056 with the “elongation of very long-chain fatty acids” (UniProt:ASU_11627, GO:0016021 integral components of the membrane), GS_09942 as “sperm-specific protein” (UniProt:ASU_04668, PROSITE:PS50202) and GS_11251 as “Uncharacterized protein,” or with the InterProScan classification, “rhodopsin-like G protein-coupled receptors (GPCRs)” (UniProt:ASU_01462, GO:0016021 integral components of membrane) (Figure 7E). The one gene lacking thorough characterization, L3E_01572, is annotated only with the associated GO-term (UniProt:Bm1034, GO:0016021 integral component of membrane). The overall gene regulation, based on maximal fold change values, of *S. scrofa* genes connected to the four *A. suum* genes was diverse as illustrated in Figure 7D. In addition to node degrees, the four *A. suum* genes are further emphasized by the fact that they participate in the four largest clusters of GEN1 identified by MCODE (Cluster 1, 2, 4, and 5, Figure 7B; Table S2 in Supplementary Material). Those four clusters are dominated by *S. scrofa* genes except for one *A. suum* gene each. The network data links the parasite’s fatty acid biosynthesis with host cluster 1, which is comprised of mostly repressed genes like Cystatin E/M and macrophage inhibitory factor 1 (MIF), known to be induced by bacterial invaders (97), and ribosomal proteins (RPS21, RPS16, RPL13, RPL18, RPL8) involved in peptide and protein synthesis (Table S2 in Supplementary Material). GO profiling of *S. scrofa* DEGs per cluster revealed significantly over-represented terms (two-sided Fisher’s Exact test, *p* < 0.05) in cluster 1 (Figures 7B,C; Figure S3 in Supplementary Material) associated with structural molecule activity (MF), cell, cell part and macromolecular complex (CC). In parallel, the sperm-specific protein of *A. suum* interacted with cluster 2 that contained mostly repressed genes for cellular and metabolic processes (BP). A dominant activation of genes was found in cluster 5 with the interspecies link to GS_11251 (*A. suum*) that shares structural similarity to known rhodopsin-like G protein-coupled receptors according to InterPro. GPCRs transduce extracellular signals through interaction with guanine nucleotide-binding (G) proteins, highlighting its potential for interspecies linkage.

Even though more extensive interpretations are limited for the DEGs that have no functional information assigned, the prominent appearance of the four *A. suum* genes in the GENs and clusters render them into attractive and suitable candidates for targeted approaches.

## Data Deposition

All sequencing data generated in this project are available from the NCBI Sequence Read Archive (SRA) and collectively available *via* the BioProject: PRJNA450204.

All analysis code is provided at https://gitlab.com/mkuhring/project_asuum.

## Author Contributions

Conceptualization: FE, BR, and SH. Investigation: FE, MK, AR, and AM. Formal analysis: MK and BR. Visualization: FE and MK. Writing—original draft: FE and MK. Writing—review and editing: FE, AM, MK, AR, BR, and SH. Funding acquisition: SH and BR. Resources: AM and AR. Supervision: SH and BR.

## Conflict of Interest Statement

The authors declare that the research was conducted in the absence of any commercial or financial relationships that could be construed as a potential conflict of interest.

## Footnotes

^1^https://gitlab.com/RKIBioinformaticsPipelines/QCumber (Accessed: March 17, 2018).

^2^https://www.bioinformatics.babraham.ac.uk/projects/fastqc/ (Accessed: March 17, 2018).

^3^https://github.com/TransDecoder/TransDecoder (Accessed: March 17, 2018).

^4^http://bioconductor.org/packages/KEGGREST/ (Accessed: March 17, 2018).

^5^http://bioconductor.org/packages/GO.db/ (Accessed: March 17, 2018).

## Supplementary Material

Table S1 Differentially expressed genes pig and worm total.

Table S2 Co-expression analysis: GEN nodes, GEN edges, GEN cluster.

Table S3 Gene ontology (GO) term declination for GO filters.

Table S4 Genome of *Ascaris suum* L3 and IPEC-J2.
