# Supplementary material for: Silent Witness: Dual-Species Transcriptomics Reveals Epithelial Immunological Quiescence to Helminth Larval Encounter and Fostered Larval Development
Source: Front Immunol. 2018 Aug 15;9:1868. doi: 10.3389/fimmu.2018.01868 (PMC6104121; doi:10.3389/fimmu.2018.01868)
Supplement: Supplementary file 5 [file presentation_1.pdf]

Supplementary Material to:

Silent witness: Dual-species transcriptomics reveals epithelial immunological quiescence to helminth larval encounter and fostered larval development

Friederike Ebner[1,*], Mathias Kuhring[2,3,4,5], Aleksandar Radonić[6], Ankur Midha[1], Bernhard Y. Renard[2], Susanne Hartmann[1]

[1]Institute of Immunology, Department of Veterinary Medicine, Freie Universität Berlin, Berlin, Germany

[2]Bioinformatics Unit (MF1), Department for Methods Development and Research Infrastructure, Robert Koch Institute, Berlin, Germany

[3]Core Unit Bioinformatics, Berlin Institute of Health (BIH), Berlin, Germany;

[4]Berlin Institute of Health Metabolomics Platform, Berlin Institute of Health (BIH), Berlin, Germany

[5]Max Delbrück Center (MDC) for Molecular Medicine, Berlin, Germany

[6]Centre for Biological Threats and Special Pathogens: Highly Pathogenic Viruses (ZBS 1), Robert Koch Institute, Seestr. 10, 13353 Berlin, Germany

* Correspondence:
Friederike.Ebner@fu-berlin.de

Supplementary Figures S1-S5

S1

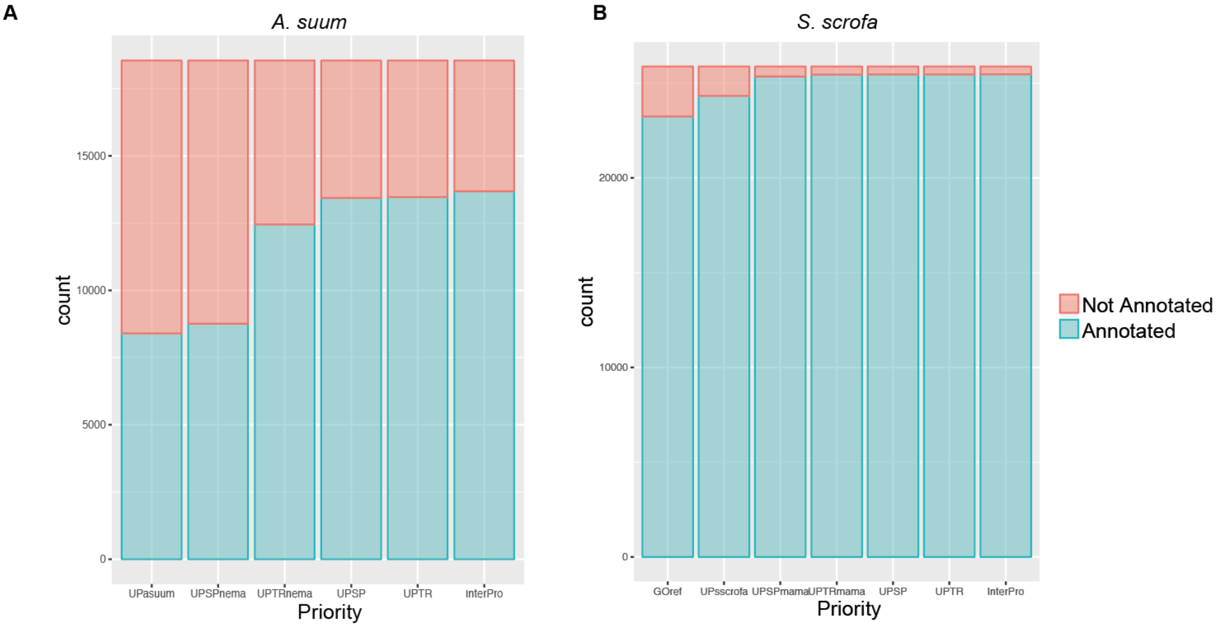

S1: Annotation yields by iterative strategy for RNA-Seq reference transcripts.
A. suum (**A**) and *S. scrofa* (**B**) UP = UniProt, SP = SwissProt, TR = TrEMBL, GOref = GO reference via Ensembl, number of transcripts being annotated (blue) after each step of the iterative annotation strategy. Left to right x-axis indicates order of database analysis (priority).

S2

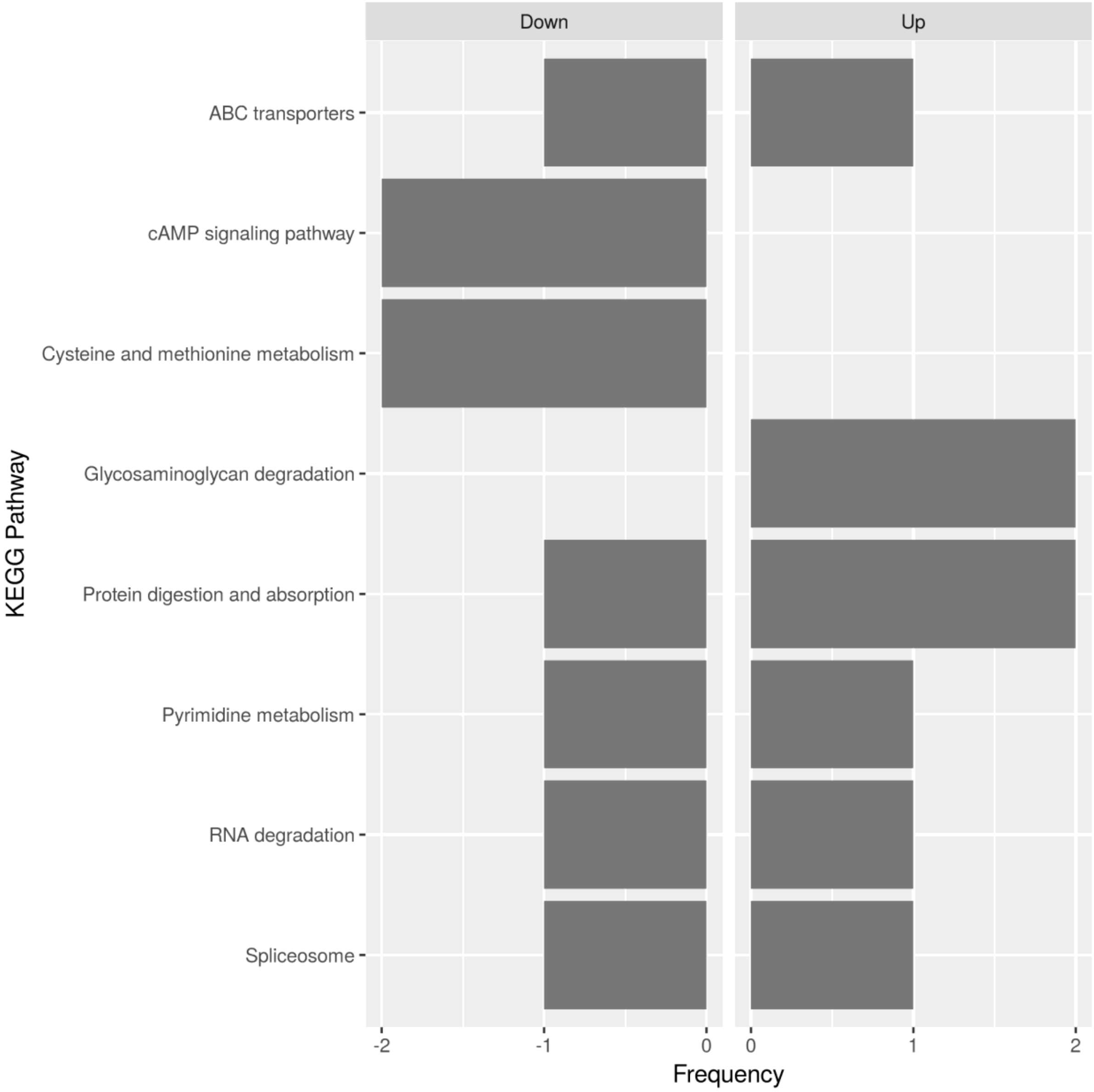

S2: Annotated KEGG pathways of *A. suum* DEGs.
Sequences were annotated with KEGG Pathways and pathways with 2 or more assigned DEGs are visualized with respect to regulation direction (based on the maximal pairwise fold-change). No pathway was significantly enriched according to enrichment analysis (Hypergeometric test, $p < 0.05$).

S3

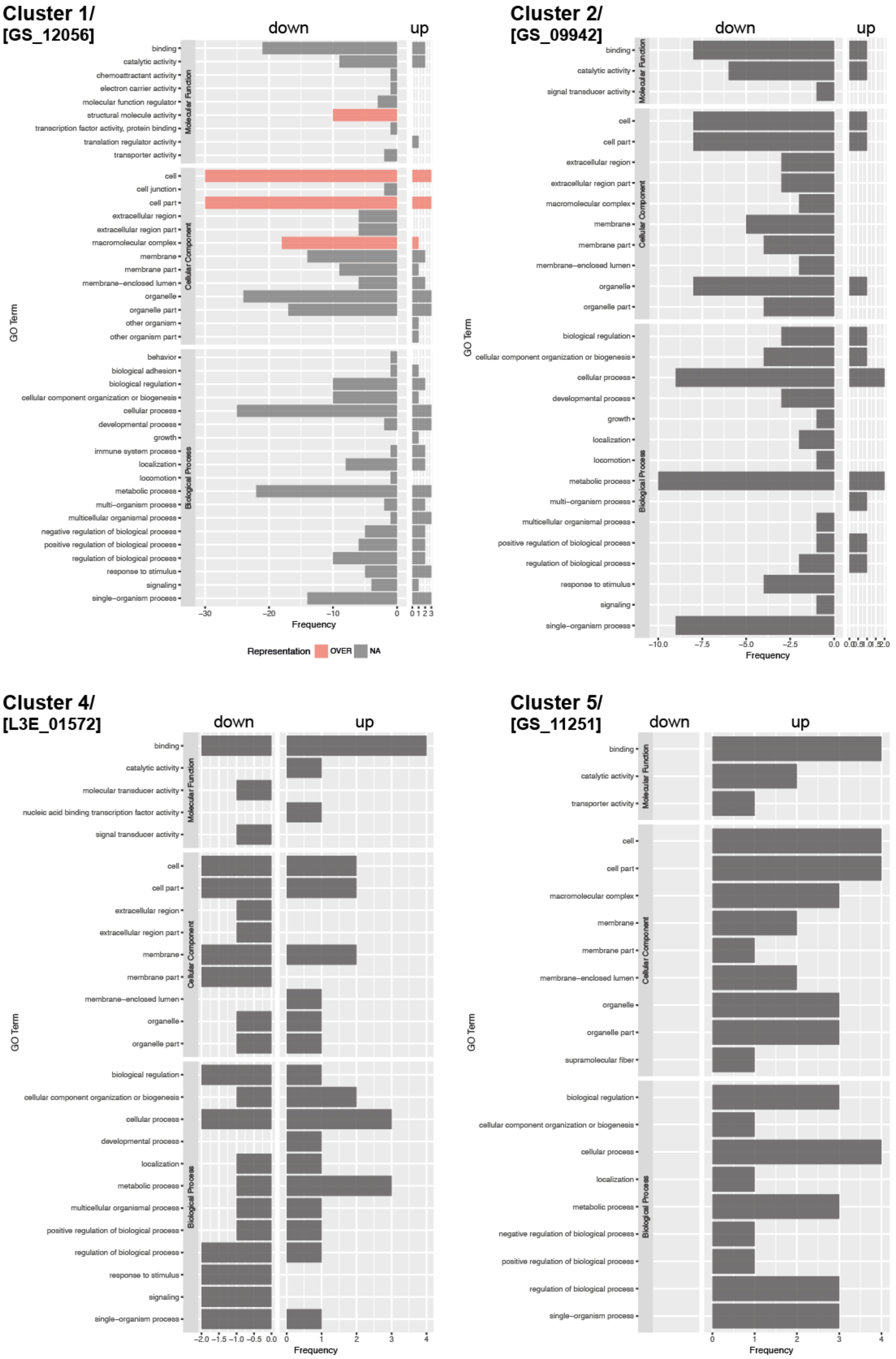

S3: GO profiles of cluster 1, 2, 4 and 5.

Level 2 GO annotation of *S. scrofa* genes (IPEC-J2 genes) of the four largest cluster in the gene co-expression network GEN1. With the cluster interacting *A. suum* genes are indicated in brackets, respectively. Significantly over- represented GO terms determined by the two-sided Fisher's Exact test in Blast2GO (adjusted $p < 0.05$) are colored in red.

S4

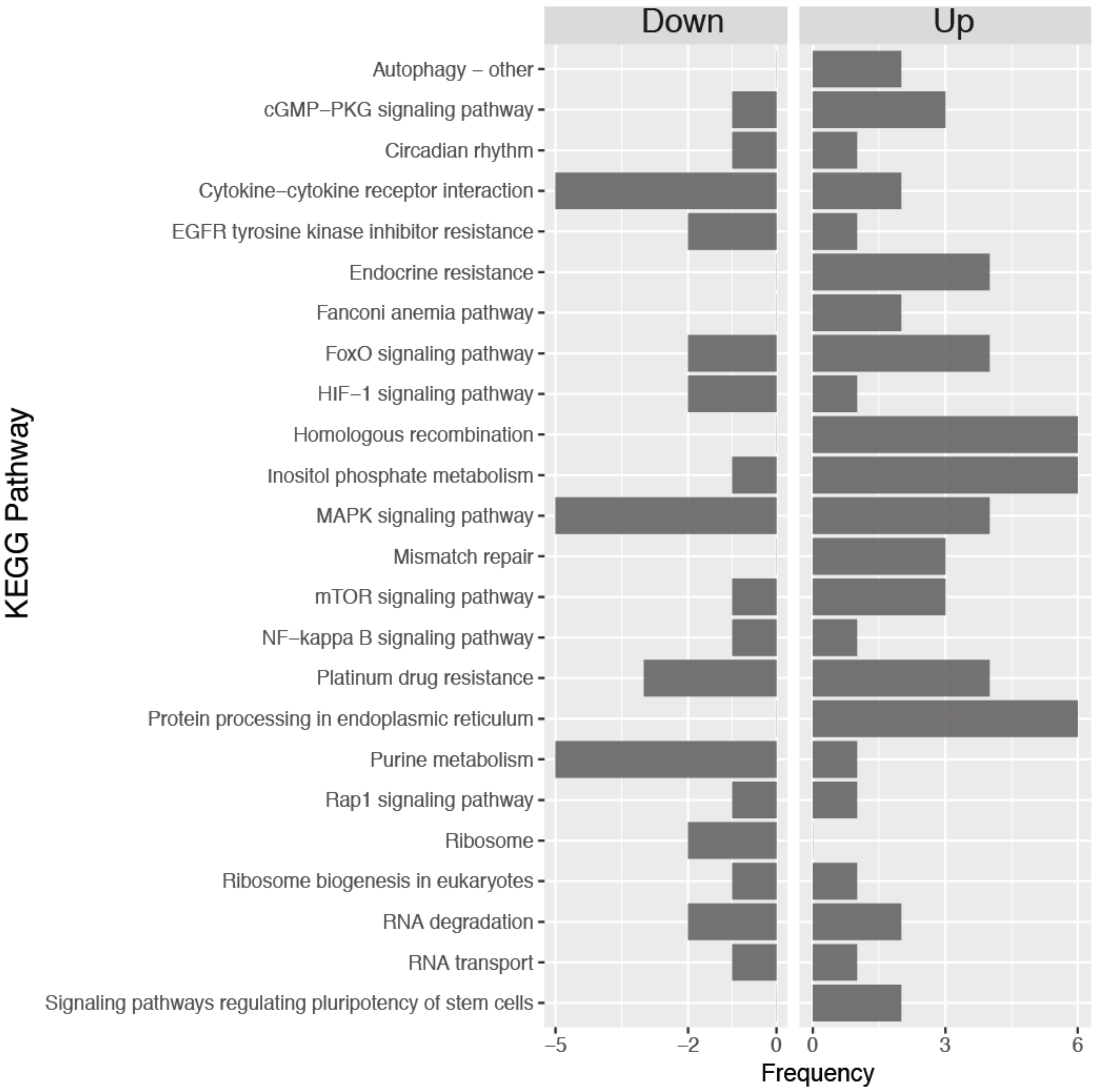

S4: KEGG pathways of *Sus scrofa* stress response

Sequences were annotated with KEGG pathways and pathways with 2 or more assigned DEGs are visualized.

S5

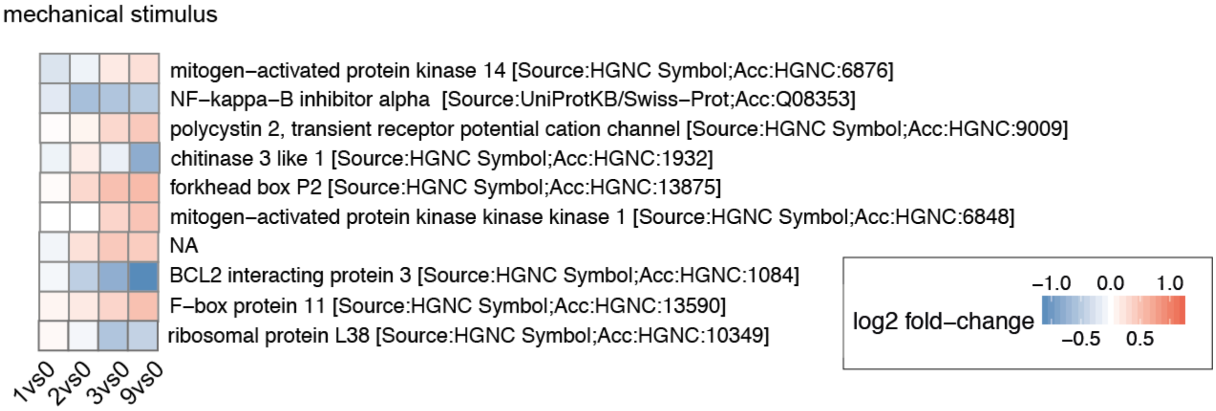

S5: Mechanically-induced *Sus scrofa* stress response
Time-resolved expression of n=10 *S. scofa* transcripts associated with mechanical stress response (mechanical stimulus).

S6

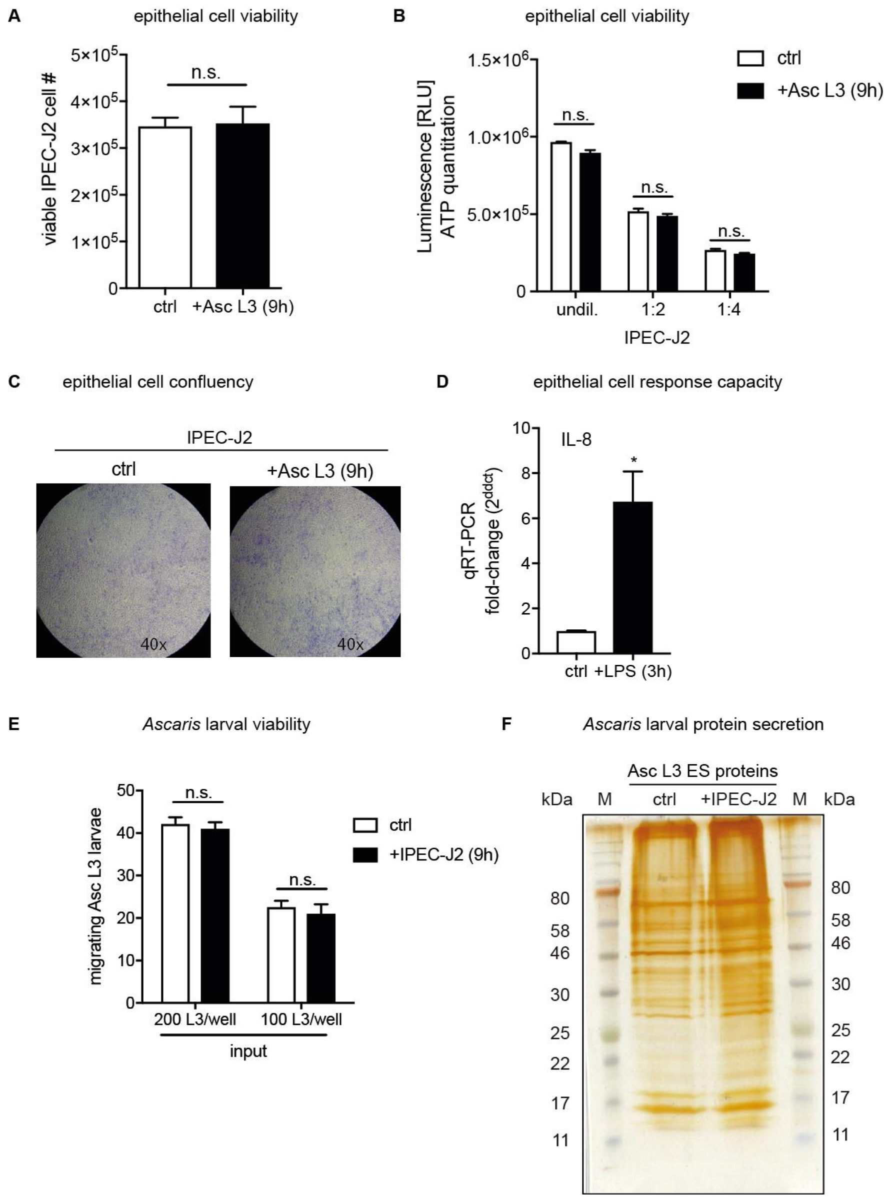

S6: Epithelial cell and *Ascaris* L3 viability and activation capacity.

Co-culture experiments were performed in 24-well format with cell to larvae ratio of 10:1 for 9h. A) IPEC-J2 cells were trypsinized from the culture dishes (n=3 per condition), washed, stained with 0.4% Trypan Blue Solution (Thermo Fisher Scientific) and counted using Neubauer improved cell counting chamber. Cell numbers of epithelial mono-cultures (ctrl) are compared to epithelial cell numbers after 9h of larval co-culture (+Asc L3 (9h)). Mann–Whitney test. B) Measuring ATP as indication for metabolically active cells using CellTiter-Glo® Luminescent Cell Viability Assay (Promega) and recovered epithelial cells from A) in 3 different dilutions (n=3 per dilution and condition, Mann–Whitney test). C) Giemsa staining (Sigma) comparing epithelial cells without larval contact (ctrl) to IPEC-J2 cells after 9h of Asc L3 co-culture. Representative pictures of n=6 separate mono-and co-cultures. D) IPEC-J2 were stimulated with LPS from *S. typhimurium* (Sigma, L6143) at 1µg/ml for 3h (n=4) and compared to unstimulated control cells (ctrl, n=4) for IL-8 transcript expression using the following primers: housekeeping gene *cycloPA* (for: CCTGAACATACGGGTCCTG, rev: AACTGGGAACCGTTTGTGTTG) and *IL-8* (for: TTCGATGCCAGTGCATAAATA, rev: CTGTACAACCTTCTGCACCCA), Light cycler 480 SYBR Green I Master mix and Light cycler 480 II instrument (Roche Life science). Relative gene expression is given in fold change compared to ctrl (2^ddct). Mann–Whitney test. E) Agar-based larval migration assay on 2 different larval input

numbers. Larvae were embedded in 0.75% agar, overlaid with BSS media and incubated over night. The numbers of larvae that migrated out of the agar were counted and compared between *Ascaris* L3 that have been co-cultured for 9h on epithelial cells (+IPEC-J2 (9h), n=7, Student's *t*-test.) *vs.* mono-cultured larvae (ctrl, n=7). F) Larval ES protein secretion. *Ascaris* larvae recovered from either mono-cultures (ctrl) or co-incubated on epithelial cells (+IPEC-J2 (9h)) were washed and cultured in fresh BSS media for another 48h. The supernatants of those follow-up cultures were collected and 2ml were TCA precipitated from each supernatant, re-suspended in SDS buffer and visualized on a silver-stained 14% SDS gel.

S7

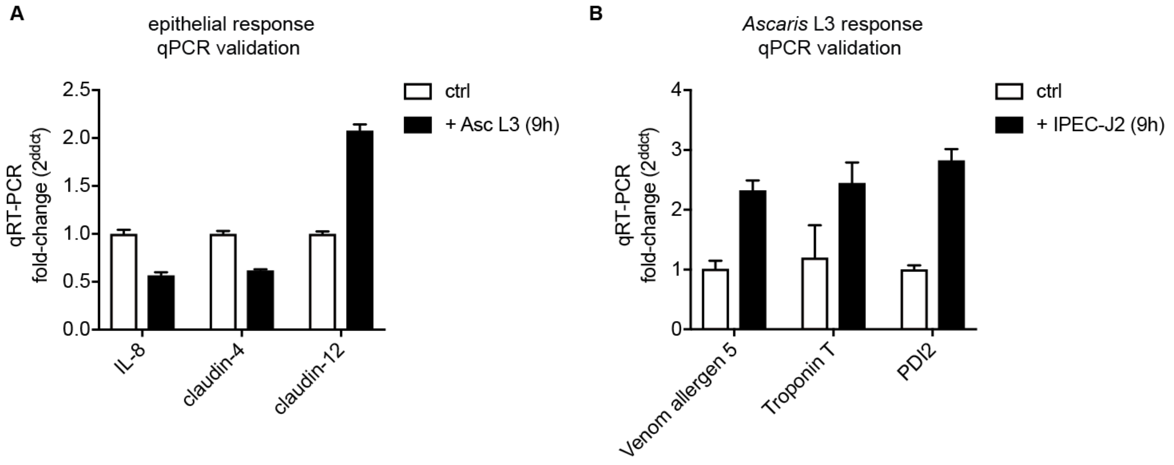

S7: qRT-PCR validation of epithelial and parasite response.

IPEC-J2 – Asc L3 co-culture experiments were performed with a cell to larvae ratio of 10:1 for 9h. Relative gene expression is given in fold change compared to ctrl (2^ddct). A) IPEC-J2 gene expression was analyzed for IL-8, claudin-4 and claudin-12 using the following primers: housekeeping gene *cycloPA* (for: CCTGAACATACGGGTCCTG, rev: AACTGGGAACCGTTTGTGTTG), target genes *il-8* (for: TTCGATGCCAGTGCATAAATA, rev: CTGTACAACCTTCTGCACCCA), *claudin-4* (for: CAACTGCGTGGATGATGAGA, rev: CCAGGGGATTGTAGAAGTCG) and *claudin-12* (for: ACCTCAAAGATCCGACAGGC, rev: GCGATTCCACACAGGAAGGA). ctrl n=3, +AscL3 (9h) n=3. B) Ascaris L3 gene expression was analyzed for venom allergen 5, Troponin T and protein disulfide isomerase 2 (PDI2) using the following primers: mean of the housekeeping genes *cybs* (for: GCCGGAGCAACGTCGGCAGCAG, rev: CTGGAATGAGTGGTACCATCGCA) and *asep36* (for: CGGTTGTATCGACGGACTTT, rev: TGAGGCTTTGACGTTCAGTG), target genes *vena5* (for: AAAGGCCAACCACTAGACGG, rev: TGCGGCTTTCAGGAGTGAAT), *tropt* (for: GGCACGCAACAAAGCACTTA, rev: TACTCGCCACCGAAACCTTC) and *pdi2* (for: TTCCGATTCTCGATGTGCCC, rev: GCTTCAGCGGTTCGTTCATC). Relative gene expression is given in fold change compared to ctrl (2^ddct). ctrl n=3; +IPEC-J2 (9h) n=4. Light cycler 480 SYBR Green I Master mix and Light cycler 480 II instrument (Roche Life science).
